# Decoding the mechanisms of allostery

**DOI:** 10.7554/eLife.88749

**Published:** 2023-06-02

**Authors:** Saif Khan, Cornelius Gati

**Affiliations:** 1 Bridge Institute, USC Michelson Center for Convergent Biosciences, Department of Biological Sciences, University of Southern California Los Angeles United States

**Keywords:** allostery, drug discovery, molecular dynamics, molecular pharmacology, muscarinic acetylcholine receptors, structural biology, None

## Abstract

A complex interplay between structure, conformational dynamics and pharmacology defines distant regulation of G protein-coupled receptors.

**Related research article** Vuckovic Z, Wang J, Pham V, Mobbs JI, Belousoff MJ, Bhattarai A, Burger WAC, Thompson G, Yeasmin M, Leach K, van der Westhuizen ET, Khajehali E, Liang YL, Glukhova A, Wootten D, Lindsley CW, Tobin AB, Sexton PM, Danev R, Valant C, Miao Y, Christopoulos A, Thal DM. 2023. Pharmacological hallmarks of allostery at the M4 muscarinic receptor elucidated through structure and dynamics. *eLife* **12**:e83477. doi: 10.7554/eLife.83477.

G protein-coupled receptors are transmembrane proteins that help to regulate a wide array of biological processes, which makes them important drug targets. However, different receptors often share a high similarity in their sequences, especially at their binding sites, which often results in challenges to develop drugs that target a specific receptor (Vuckovic et al., 2019; Singh and Karnik, 2021).

For example, the five members of a muscarinic acetylcholine receptor subfamily (M_1_-M_5_ mAChR) have essential roles in various physiological processes (Wess et al., 2007). In particular, M_4_ mAChR is of major therapeutic interest due to its involvement in regulating dopaminergic neurons involved in cognition, psychosis and addiction, while others, such as M_1_ mAChR, can be targeted to treat cognitive decline in Alzheimer’s disease (Wess et al., 2007). However, these receptors share highly similar binding sites, and drugs that target a particular mAChR receptor often inadvertently activate other receptors in the subfamily, thereby causing adverse side effects (Felder et al., 2018).

As an alternative to targeting the primary binding site on the receptor (also known as the orthosteric site) with a drug, it is sometimes possible to regulate a receptor by targeting a remote (or allosteric) site. Since there is much less similarity in the sequences of allosteric sites, this approach makes it possible to design highly selective drugs with reduced side effects.

Now, in eLife, David Thal, Arthur Christopoulus and Celine Valant (all at Monash University), Yinglong Miao (University of Kansas) and colleagues – including Ziva Vuckovic, Vi Pham and Jesse Mobbs (all at Monash) and Jinan Wang (Kansas) as joint first authors, along with colleagues in Japan, the United Kingdom and the United States – report on the molecular mechanisms that govern allostery in human M_4_ AchR (Vuckovic et al., 2023). The researchers used two ligands that targeted the orthosteric site (acetylcholine and iperoxo), and two positive modulators that targeted the allosteric site (VU154 and LY298). Both modulators have shown antipsychotic efficacy in preclinical rodent models, but these results have failed to translate into human studies (Suratman et al., 2011; Dupuis et al., 2010). Nevertheless, they remain useful tools for investigating allostery in G protein-coupled receptors (Bubser et al., 2014).

Vuckovic et al. used two types of biochemical assays to determine the pharmacological characteristics of the allosteric modulators. This revealed that both LY298 and VU154 display a phenomenon called ‘probe dependence’, meaning that they had a stronger effect when the orthosteric ligand was acetylcholine rather than iperoxo. They also showed that these effects were caused by an increase in the binding affinities of the orthosteric ligands, rather than by any modulation of signaling through the receptor (Figure 1A). Moreover, LY298 was the more potent modulator as it caused a 400-fold increase in binding affinity, compared with a modest 40-fold increase for VU154.

**Figure 1.**
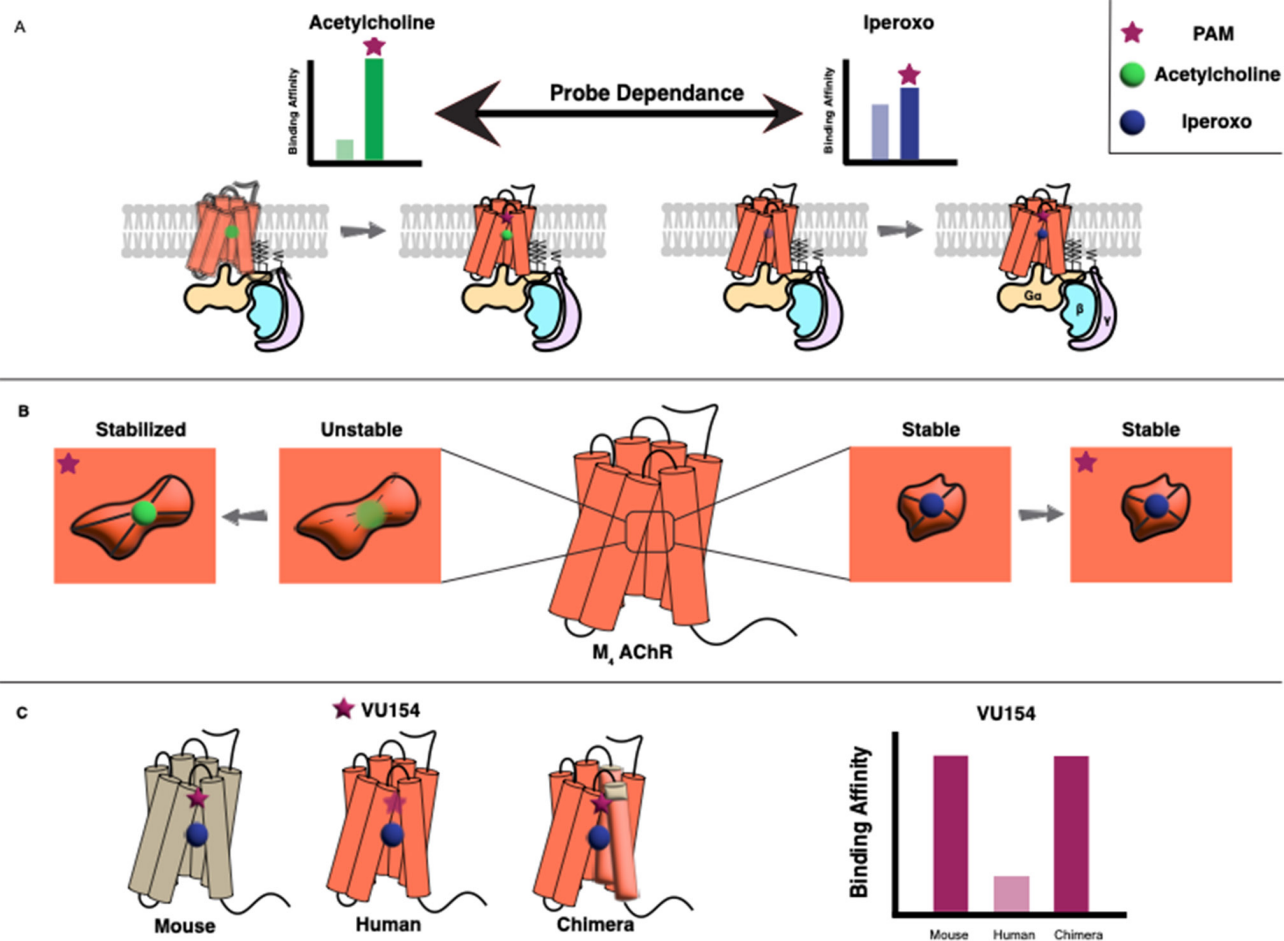
Allosteric regulation of G protein-coupled receptors. (**A**) G protein-coupled receptors (GPCRs) are transmembrane proteins (bottom) that can be regulated by orthosteric ligands (such as acetylcholine or iperoxo) and positive allosteric modulators (PAMs). Vuckovic et al. studied a receptor called M_4_ AChR and found that two PAMs (VU154 or LY298; purple stars) bind more tightly when the orthosteric ligand is acetylcholine (green circle), and less tightly when it is iperoxo (blue circle); this is referred to as “probe dependance”. (**B**) This probe dependence can be explained by differences in the binding of acetylcholine and iperoxo at the orthosteric site, which is inside a ‘pocket’. This pocket contracts around iperoxo, which results in iperoxo and the allosteric ligand forming a stable complex, but it does not contract around acetylcholine, which results in a more dynamic complex. (**C**) The ability of VU154 to bind to the receptor varies from species to species and is higher in mice compared to humans. However, introducing mutations to make the genetic sequence in the human receptor more like that of the mouse receptor led to an increase in binding affinity.

To uncover the molecular basis for these results, Vuckovic et al. used cryogenic electron microscopy (cryoEM) to obtain four structures of M_4_ AChR bound to its cognate G protein and in complex with various ligands. The structural analyses – combined with molecular dynamics simulations – enabled the authors to uncover the underlying dynamics and conformational changes that are otherwise missed through static snapshots of cryoEM structures.

The experiments revealed that the allosteric sites for both VU154 and LY298 were, as expected, located in a region of the receptor called the extracellular vestibule. The orthosteric sites overlapped with those in other members of the mAChR subfamily and were located inside a central ‘pocket’ in the receptor; However, it was noticed that this pocket was contracted around iperoxo but not around acetylcholine. The smaller binding pocket, along with the rotation of a specific tyrosine residue, resulted in more stable interactions for iperoxo within the orthosteric site. On the other hand, the binding of acetylcholine was seen to be more dynamic with fewer stable interactions (Figure 1B).

Surprisingly, even though iperoxo bound to the receptor more tightly than acetylcholine, its ability to promote signaling through the receptor was lower. Vuckovic et al. suggest that since the acetylcholine-bound M_4_ AChR is more dynamic, it can sample a large range of conformations, including those that couple to and activate G protein. This allows the receptor to efficiently activate the G protein and increase the signaling response.

The structures and molecular dynamics simulations also helped uncover the molecular basis for the probe dependence of the allosteric modulators. It is possible that both had stronger effects on the acetylcholine-bound receptor due to the stabilization of an inherently dynamic structure. Conversely, since the iperoxo-bound receptor was already very stable, the modulatory effects were negligible. This result provides a key future consideration for the development of allosteric drugs that target G protein-coupled receptors.

Using mutational studies, Vuckovic et al. also identified a network of amino acids that were important to the conformational dynamics of the protein, some of which showed maximum variability between structures and modulated the signaling efficacy of both orthosteric and allosteric ligands.

Lastly, the researchers investigated why VU154 is potent in some species but not in others. Based on their initial findings, VU154 was a weaker positive allosteric modulator than LY298 in humans because it poorly stabilized the active receptor conformation. However, its effects in mice were stronger, and were comparable to the effects of LY298 in humans. Using mutational studies, Vuckovic et al. identified three important residues on the human receptor that confer species-selectivity. Mutating these to the equivalent residues in the mouse sequence resulted in improved allostery by VU154 in functional studies and stable binding in simulations.

In conclusion, Vuckovic et al. have described the complex interplay between structure, conformational dynamics and pharmacology that defines allostery at G protein-coupled receptors. Their work provides a detailed framework to guide future drug discovery efforts focused on the muscarinic receptor subfamily.
